# Induction Effect of Fluorine-Grafted Polymer-Based Electrolytes for High-Performance Lithium Metal Batteries

**DOI:** 10.1007/s40820-025-01738-9

**Published:** 2025-05-13

**Authors:** Haiman Hu, Jiajia Li, Fei Lin, Jiaqi Huang, Huaiyang Zheng, Haitao Zhang, Xiaoyan Ji

**Affiliations:** 1 Energy Engineering, Division of Energy Science, Luleå University of Technology, 97187 Luleå, Sweden; 2 CAS Key Laboratory of Green Process and Engineering, Beijing Key Laboratory of Ionic Liquids Clean Process, Institute of Process Engineering, Chinese Academy of Sciences, Beijing, 100190 People’s Republic of China

**Keywords:** Fluorine-grafted polymer, Induction effect, High interface stability, Quasi-solid-state electrolytes, Lithium metal battery

## Abstract

Quasi-solid-state composite electrolytes (QSCEs) show promise for high-performance solid-state batteries, while they still struggle with interfacial stability and cycling performance. Herein, a F-grafted QSCE (F-QSCE) was developed via copolymerizing the F monomers and ionic liquid monomers. The F-QSCE demonstrates better overall performance, such as high ionic conductivity of 1.21 mS cm^–1^ at 25 °C, wide electrochemical windows of 5.20 V, and stable cycling stability for Li//Li symmetric cells over 4000 h. This is attributed to the significant electronegativity difference between C and F in the fluorinated chain (‒CF_2_‒CF‒CF_3_), which causes the electron cloud to shift toward the F atom, surrounding it with a negative charge and producing the inductive effect. Furthermore, the interactions between Li^+^ and F, TFSI^‒^, and C are enhanced, reducing ion pair aggregation (Li^+^‒TFSI^‒^‒Li^+^) and promoting Li^+^ transport. Besides, ‒CF_2_‒CF‒CF_3_ decomposes to form LiF preferentially over TFSI^–^, resulting in better interfacial stability for F-QSCE. This work provides a pathway to enable the development of high-performance Li metal batteries.

**Supplementary Information:**

The online version contains supplementary material available at 10.1007/s40820-025-01738-9.

## Highlights

- Fluorine-grafted quasi-solid-state composite electrolyte (F-QSCE)@30 exhibits high ionic conductivity of 1.21 mS cm^–1^ at 25 °C.
- The inductive effect weakens the coordination between Li^+^ and TFSI^‒^, enhancing Li^+^ transport.
- LiF in the solid electrolyte interphase of F-QSCE@30 comes from decomposed F segments, not TFSI^‒^.
- F-QSCE@30 maintains stability with Li metal for over 4000 h and inhibits dendrite growth.

**Supplementary Information:**

The online version contains supplementary material available at 10.1007/s40820-025-01738-9.

## Graphical abstract

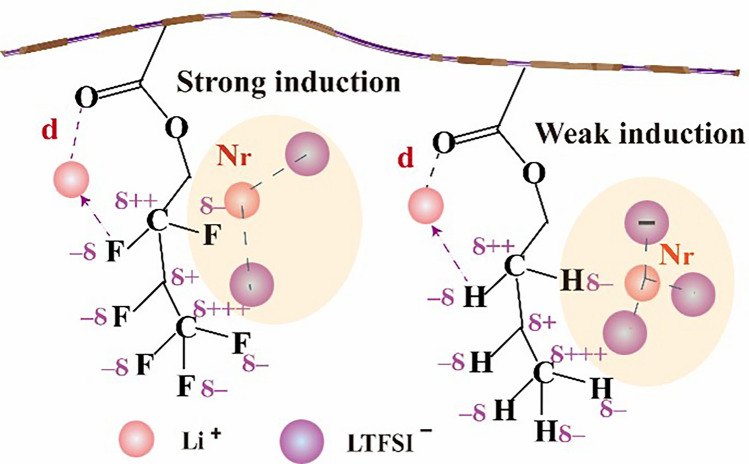

## Introduction

With the rising need for energy and electric vehicles, developing high-performance and safe batteries has become particularly important [1]. Lithium metal, known for its remarkable specific capacity (3860 mAh g^–1^) [2–7], low density (0.53 g cm^–3^), and electrochemical potential (− 3.04 V vs H^+^/H_2_) [8], is widely considered a promising anode for advancing high-performance Li metal batteries (LMBs) [9–12]. Despite the significant advantages of the Li metal itself, LMBs, based on commercial organic electrolytes, still present safety challenges linked to liquid leakage and high flammability [13, 14]. These issues have driven the rise of composite solid electrolytes (CSEs), which consist of a polymer backbone, filler, and Li salt. With their enhanced safety, superior thermal stability, and scalability, CSEs are regarded as potential candidates for future solid-state electrolytes [15]. However, inherent challenges, including low ionic conductivity and unstable interface, continue to limit the performance of CSEs [9, 16].

To address the challenges of CSEs, several strategies have been developed and implemented. For instance, the in situ polymerization strategy has been widely adopted to improve interfacial contact by forming a seamless electrode–electrolyte interface, while also enhancing the ionic conductivity [17, 18]. Adding buffer layers between the electrolyte and electrode has proven effective in mitigating interfacial reactions and reducing dendrite growth [19]. However, the added buffer layer also hinders the conduction of ions. Other approaches, such as electrolyte surface treatment and increasing stacking pressure, are simple modification methods, offering limited improvements. More advanced strategies, including constructing interfacial transition layers [20] and composite anodes, have shown promise in reducing interfacial resistance and enhancing cycling performance. However, these methods are often complex and costly, accompanied by insufficiently high ionic conductivity [21]. While these advancements have addressed some challenges of CSEs, further optimization is needed.

Quasi-solid-state composite electrolytes (QSCEs) incorporate a small amount of liquid into CSEs, offering the high ionic conductivity of liquids alongside the improved thermal stability, safety, and scalability of CSEs [15]. Although this combination improves the overall performance of the electrolytes compared to the traditional CSEs, it still fails to meet the performance requirements for applications, calling for further development [9]. Adding more liquids has been proposed to further develop, where the novel ionic liquids (ILs) have been extensively studied. The addition of ILs with high chemical stability and ionic conductivity (e.g., 1-ethyl-3-methylimidazolium bis(trifluoromethylsulfonyl)imide (EMIMTFSI) [22] and n-propyl-n-methylpyrrolidinium bis(trifluoromethanesulfonyl)imide (PYR_13_TFSI) [23]) can expand the electrochemical stability window and improve interfacial stability with the lithium metal [24]. However, to achieve sufficient ionic conductivity (> 1.00 mS cm^–1^ at 25 °C), [25] the liquid content typically needs to be more than 30 wt% of the total weight of the QSCEs [26], which can significantly compromise the mechanical strength, diminishing the ability to suppress the lithium dendrite growth and ultimately resulting in short circuits and battery failure [27, 28]. Therefore, adding more liquid to enhance the overall performance of QSCEs is inadequate.

It is well known that within QSCEs, the polymer backbone plays a critical role in determining their interfacial stability as well as ionic conductivity [28]. For instance, polymers, including polyethylene oxide (PEO) and polyethylene glycol methyl ether acrylate (PEGMA), [29] can generate a stable interface with the Li metal, but their ionic conductivity tends to be low [30, 31]. Therefore, designing and modifying polymers to provide stable interfaces and enhance their ionic conductivity have been proposed [32], where several strategies, including polymer blending/cross-linking [33] and the incorporation of highly electronegative elements [34] into the polymer matrix, have been investigated. It was found that blending and cross-linking can moderately improve the ionic conductivity of QSCEs [35], but cannot achieve high interfacial stability with the Li metal; the introduction of highly electronegative elements into the polymer, especially the F atom with the highest electronegativity, will change the electron density of the chain segments, producing an inductive effect [36], which can adjust interactions between the highly electronegative atoms and Li^+^, thus affecting the ionic conductivity [36]. Generally, it is also believed that introducing the F elements helps generate a stable solid electrolyte interphase (SEI), enhancing compatibility with the Li metal [34]. For example, Tang et al. [37] developed a fluorinated solid polymer electrolyte using a fluorinated cross-linker, achieving an ionic conductivity of 1.37 mS cm^–1^ at 25 °C and enhancing electrochemical stability, due to the strong electron-withdrawing inductive effect of the F segments and the formation of a LiF-rich SEI. However, the performance of either Li//Li cell (2500 h at 0.1 mA cm^‒2^) or full cell (LiNi_0.6_Co_0.2_Mn_0.2_O_2_//Li, 200 cycles at 0.5C) is still insufficient. Similarly, Lin et al. [38] designed an innovative fluorinated electrolyte framework, but the ionic conductivity (0.04 mS cm^–1^, at 25 °C) is low and the cycling is inadequate (2600 h; 2.50 –4.50 V, < 100 cycles with the high-voltage LiCoO_2_). These results indicate that developing fluorine (F)-grafted QSCE is promising, and the F segments play an important role in improving the ionic conductivity and promoting the formation of a LiF-rich SEI layer, thereby achieving long cycling of the battery. However, the interfacial stability and cycling performance of the currently developed F-grafted QSCEs are not yet fully optimized due to several inherent limitations. Firstly, while the fluorinated chains enhance ionic conductivity, the overall ionic conductivity of the polymer matrix remains relatively low compared to liquid electrolytes, primarily due to the restricted mobility of Li^+^ within the polymer framework. Secondly, although the fluorinated segments improve interfacial stability by promoting the formation of a LiF-rich SEI layer, the polymer backbone itself is still susceptible to decomposition at high voltages or during prolonged cycling. Besides, the reduction of LiTFSI at the electrode interface cannot be effectively suppressed, leading to the generation of undesirable by-products that degrade the SEI layer over time [39]. These factors collectively limit the long-term cycling performance and interfacial stability of the F-grafted QSCEs, highlighting the need for further improvement of the performance [40, 41]. Also, the specific induction effect of the F segments is unclear, and its influence on the SEI formation mechanism still needs further study.

Herein, a F-grafted QSCE, where the fluorinated segments were grafted onto the polymer backbone to form a linear polymer terminated with the F-containing segments following the O-containing functional groups, was developed to boost the overall performance of electrolytes, and the impact of the F segments on the performance as well as on the SEI composition and formation was investigated. To achieve this, hexafluorobutyl methacrylate (HFM) was chosen as the fluorinated monomer, 1-vinyl-3-butylimidazolium bis(trifluoromethylsulfonyl)imide (VBImTFSI) was selected as the flexible monomer to prepare the polymer, the IL-confined SiO_2_@IL was incorporated as a filler to improve the ionic conduction, and the glass fiber was employed as a substrate to increase the mechanical strength. The optimal ratio of these constituents was determined by evaluating their electrochemical stability window and ionic conductivity, and the identified optimal electrolyte was subjected to further investigation, including characterization, electrochemical properties, and performance, as well as the analysis of the dissociation of LiTFSI and the chemical environment of Li^+^. To illustrate the role of F, a F-free electrolyte with the same ratio of each constituent as the optimal F-QSCE was prepared, where the non-fluorinated methyl methacrylate (MMA) monomer was chosen. The research was combined with the advanced theoretical (MD simulations and DFT calculations) and experimental (the X-ray photoelectron spectroscopy (XPS) and the time-of-flight secondary ion mass spectrometry (ToF**–**SIMS)) tools.

## Experimental Section

### Materials

1-Vinyl-3-butylimidazolium bis(trifluoromethylsulfonyl)imide (VBImTFSI) and n-propyl-n-methylpyrrolidinium bis(trifluoromethanesulfonyl)imide (PYR_13_TFSI) were purchased from the Lanzhou Institute of Chemical Physics. Al foil, Li bis-trifluoromethanesulfonimide (LiTFSI, 99.95%), LiFePO_4_, LiNi_0.6_Co_0.2_Mn_0.2_O_2_ (NCM622), and super P were obtained from the MTI Corporation. Other chemicals, including mesoporous silica (SiO_2_, pore size of 6–10 nm, specific surface area of 600–800 m^2^ g^‒1^), methanol, 1-hydroxycyclohexyl phenyl ketone (photoinitiator), n-methyl pyrrolidone (NMP), diethyl carbonate (DEC), hexafluorobutyl methacrylate (HFM), and methyl methacrylate (MMA), were purchased from Aladdin. The glass fiber with a thickness of 260 µm was purchased from the Guangdong Canrd New Energy Technology Co., Ltd, China.

### Preparation of IL-Confined SiO_2_ (SiO_2_@IL)

Typically, a mixture of SiO_2_ and PYR_13_TFSI with a certain molar ratio was added to a glass bottle with 5 mL methanol. The mixture was stirred for 12 h under sealed conditions. Afterward, methanol was evaporated using a rotary evaporator. The resulting powder, referred to as SiO_2_@IL, was subsequently dried in a vacuum oven at 80 °C for 12 h and then stored for use as the fillers to prepare QSCEs.

### Preparation of Electrolytes

The electrolytes were prepared step by step.

**Step 1**: VBImTFSI and HFM with a certain molar ratio, the LiTFSI salt (20 wt% of VBImTFSI and HFM monomers), and photoinitiator (2 wt% of VBImTFSI and HFM monomer) were added into a glass bottle with 0.5 mL DEC and stirred for 5 h to obtain a uniform mixture solution. After that, 90 µL of the mixture solution was added to the glass fiber with a diameter of 16 mm and then under a UV light for 25 min to prepare the electrolytes for determining the ionic conductivity to optimize the ratio of VBImTFSI to HFM.

**Step 2**: The LiTFSI salt with different amounts (20, 30, and 40 wt% of (VBImTFSI + HFM)) was added where the ratio of VBImTFSI to HFM was fixed as the optimized one to prepare the electrolytes. After that, 5 µL PYR_13_TFSI was added to the surface of the prepared electrolytes and dried in a vacuum oven at 80 °C for 12 h to determine their ionic conductivity and electrochemical stability window in an SS//SS and SS//Li symmetrical cell to identify the optimal content of LiTFSI.

**Step 3**: Based on the optimized ratio of VBImTFSI to HFM and the optimized content of LiTFSI, the fluorinated electrolyte was prepared together with the photoinitiator and SiO_2_@IL. More specifically, VBImTFSI, HFM, LiTFSI, photoinitiator, and SiO_2_@IL were added into a glass bottle with 0.5 mL DEC and stirred for 4 h to prepare QSCE with the UV curing procedure. For comparison, MMA (F-free monomer) was used to replace HFM but kept the same ratio and procedure to prepare the F-free QSCE. After that, 5 µL of PYR_13_TFSI was added on the surface of both F-QSCE and F-free QSCE, and the prepared electrolytes were dried at 80 °C for 12 h under vacuum and stored in the glove box (Mikrouna, Universal 3660, H_2_O < 0.01 ppm and O_2_ < 0.01 ppm).

### Cathode Preparation and Cell Assembly

To prepare the cathodes, a mixture of LiFePO4 or NCM622 powder, super P, and PVDF with a weight ratio of 8:1:1 was dispersed in NMP and stirred magnetically for 12 h to form a uniform slurry. Then, the slurry was applied onto aluminum foil as the current collector and dried at 80 °C in a vacuum oven overnight to obtain the cathode. The cathode material was subsequently cut into disks with a diameter of 14 mm. The mass loading of the cathode was maintained within the range of 1.5–2.0 mg cm^‒2^. The coin cells were assembled in an argon-filled glove box, utilizing the Li metal as the anode, LiFePO4 or NCM622 as the cathode, and the prepared F-QSCE or F-free QSCE as the electrolytes with each cutting into a disk at a diameter of 16 mm.

The information on characterization, electrochemical measurements, and computer simulations is described in the Supporting Information.

## Results and Discussion

### Identifying Optimal Composition of Electrolytes and Further Characterization

**Fig. 1:**
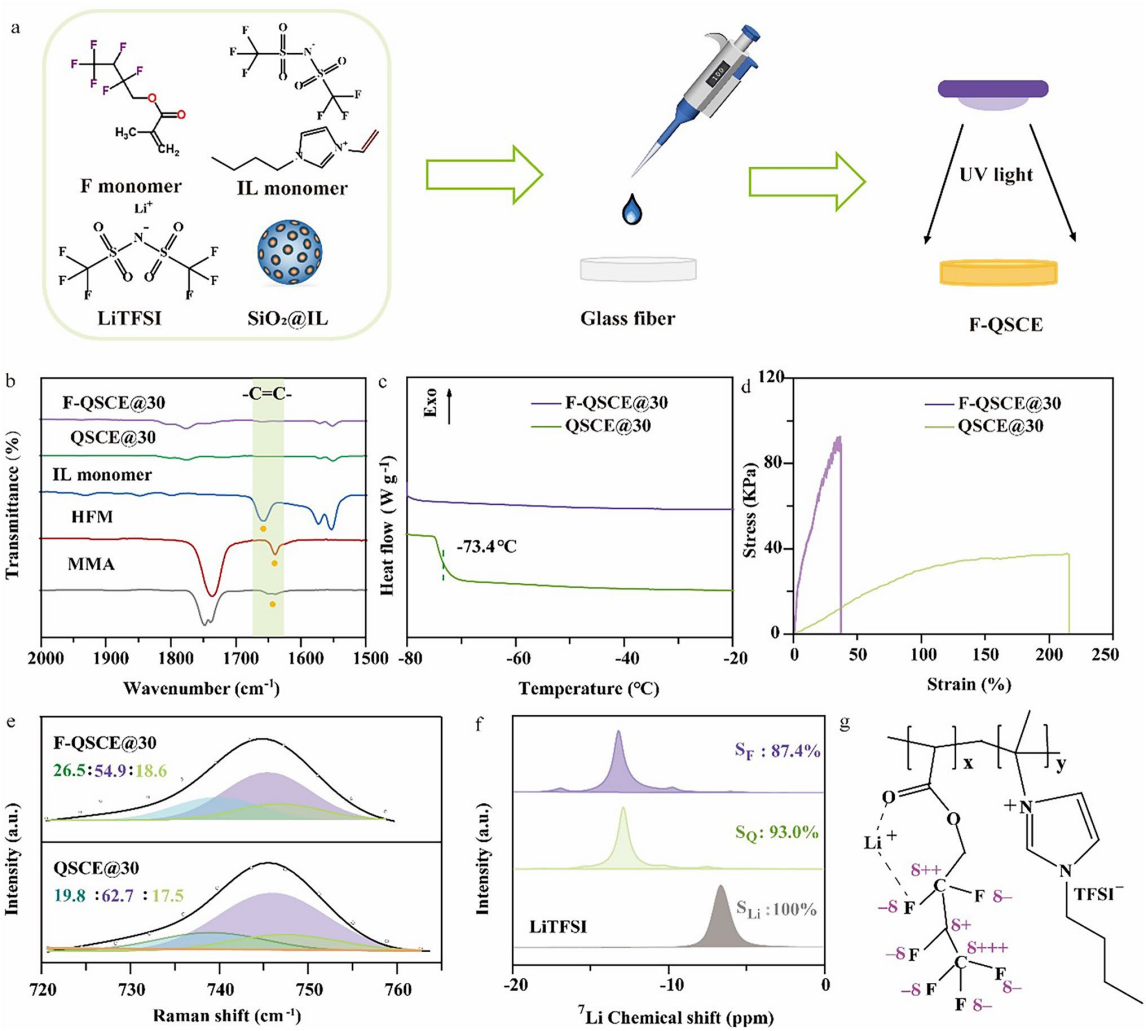
**a** Schematic illustration of the preparation of F-QSCEs. **b** FTIR spectra of MMA, HFM, IL monomer, QSCE@30, F-QSCE@30. **c** DSC curves and **d** stress–strain curves of QSCE@30 and F-QSCE@30. **e** Raman spectrum of F-QSCE@30 and QSCE@30. **f** ^7^Li NMR spectra of F-QSCE@30, QSCE@30, and LiTFSI. **g** Interaction of Li and ‒C = O‒O‒CF_2_‒ in the polymer

The fluorinated electrolytes were prepared using the method as illustrated in Fig. 1a with the details described in Sect. 2.3. Briefly, the preparation involves mixing a precursor solution of VBImTFSI, HFM, LiTFSI, SiO_2_@IL, and a photoinitiator, which is then deposited onto the glass fiber disks and cured by the UV light. Here, SiO_2_@IL was prepared from PYR_13_TFSI and SiO_2_ at a molar ratio of 23:1 based on previous work [42]. To optimize the composition, the ratio of VBImTFSI to HFM and the LiTFSI content were adjusted, and their ionic conductivities and electrochemical stability windows were determined. The ionic conductivity and specific composition of the polymer-based electrolytes are provided in Table S1. It shows that, among the electrolytes at a certain molar ratio of VBImTFSI/HFM ranging from 1:1 to 5:1, the one at 4:1 provided the highest ionic conductivity (Fig. S1), and this ratio was fixed for further study. Based on the 4:1 molar ratio of VBImTFSI/HFM, the content of LiTFSI was further optimized in a range of 20–40 wt%, and the one with 30 wt% LiTFSI exhibited the highest ionic conductivity of 0.69 mS cm^‒1^ at 25 °C (Fig. S2) and widest electrochemical stability window of 5.20 V (Fig. S3), resulting from an optimal balance between ion dissociation and mobility. More specifically, at 30 wt% LiTFSI, the electrolyte achieves a stable structure that resists decomposition at high voltages, thus providing a wide electrochemical window; however, at very high LiTFSI concentrations (e.g., 40 wt%), ion pairing may occur, reducing conductivity and narrowing the electrochemical window. Based on the above results, the optimal electrolyte was identified as the one at the 4:1 molar ratio of VBImTFSI/HFM, 30 wt% LiTFSI, together with 5 wt% SiO_2_@IL, which was designated as F-QSCE@30. According to the composition of F-QSCE@30, the fluorine-free electrolyte was prepared by replacing HFM with MMA and labeled as QSCE@30.

The Fourier transform infrared (FTIR) spectra of HFM, MMA, VBImTFSI, QSCE@30, and F-QSCE@30 were determined to confirm the successful polymerization of QSCE@30 and F-QSCE@30. The results are presented in Fig. 1b. It shows that all the used monomers of QSCEs, i.e., HFM, MMA, and VBImTFSI, present C = C bonds, and their corresponding characteristic peaks are located at 1650, 1652, and 1651 cm^–1^, respectively [37]. After the UV polymerization, no characteristic peak of C = C bonds is observed in QSCE@30 and F-QSCE@30, confirming that the monomers underwent a chemical reaction and a polymer network was successfully formed [32].

Figure 1c shows the glass transition temperature (*T*_g_) of QSCE@30 and F-QSCE@30, measured by DSC. It indicates that F-QSCE@30 exhibits a lower *T*_g_ ( < –80.0 °C) compared to –73.4 °C for QSCE@30. The lower *T*_g_ observed in F-QSCE compared to QSCE can be attributed to the introduction of fluorine, which significantly influences the polymer matrix. The high electronegativity of fluorine weakens intermolecular interactions, such as hydrogen bonding or dipole–dipole interactions, involving the C–O–C = O group. This reduction in the intermolecular interactions enhances chain mobility, leading to a lower *T*_g_. Also, the fluorine-containing groups may act as internal plasticizers, increasing free volume and further facilitating segmental motion. These combined effects result in the observed decrease in *T*_g_ [22]. A lower *T*_g_ suggests a higher degree of amorphousness in the polymer, which can enhance the ion transport and improve the ionic conductivity [32]. Therefore, F-QSCE@30 is potentially more effective in achieving efficient ion conduction.

The mechanical strength of electrolytes is essential to achieve desirable compatibility with electrodes. Figure 1d presents the stress/strain curves for both F-QSCE@30 and QSCE@30. F-QSCE@30 demonstrates a tensile strength of 92.7 kPa, significantly exceeding that of QSCE@30 (37.4 kPa) but still lower than that of the reported (Table S2), which needs to be further improved. It should also be noticed that the elongation at break for F-QSCE@30 is 37%, which is substantially lower than 212% for QSCE@30. These observed phenomena can be illustrated as follows: Incorporating the F segments enhances the interactions among the ester groups on the polymer chain, thereby increasing the tensile strength. While introducing numerous rigid –CF_3_ groups raise the rigidity of QSCE, reducing the elongation at break [43]. The high tensile strength of F-QSCE@30 will effectively inhibit the growth of Li dendrites, which will markedly improve the performance. Therefore, F-QSCE@30 is promising.

To explore the coordination environment of TFSI^‒^ and Li^+^, the Raman spectra of F-QSCE@30 and QSCE@30 were obtained (Fig. 1e), and the Gaussian–Lorentzian model was employed to deconvolute the Raman spectra. The peak observed in the lower wavenumber range (730–740 cm^‒1^) is ascribed to the free TFSI^‒^; the peaks at mid-wavenumber (745–747 cm^‒1^) correspond to the contact ion pairs (CIP), where TFSI^‒^ interacts with a single Li^+^; at higher wavenumber, the peaks refer to the aggregates (AGG), where TFSI^‒^ interacts with two or more Li^+^ [15]. Therefore, CIP and AGG are related to the TFSI^‒^ coordinated with Li^+^ (Li‒*x*TFSI), and the Li coordination number (*x*) can be determined using Eq. (1) according to the Gaussian–Lorentzian model [28]:

1 $$x = \frac{1}{{M({\text{Li}}^{ + } )}} \times \frac{{A({\text{CIP}} + {\text{AGG}})}}{{A({\text{free}}) + A({\text{CIP}} + {\text{AGG}})}}$$

where *M*(Li^+^) is the mole percentage of the Li salt, and the spectral area corresponding to the ion clusters (i.e., CIP and AGG) is represented as *A*(CIP + AGG), while the area corresponding to free TFSI^‒^ is *A*(free).

According to the peaks shown in Fig. 1e, the percentages of free TFSI^‒^ were estimated with values of 26.5% for F-QSCE@30 and 19.8% for QSCE@30. The higher the content of the free TFSI^‒^, the lower the number of “TFSI^‒^ coordinated with Li^+^,” and thus the dissociation of LiTFSI in F-QSCE@30 is higher than that in QSCE@30 [44]. Further, the Li coordination numbers, *x*, for both F-QSCE@30 and QSCE@30 were calculated using Eq. (1), derived from the data presented in Fig. 1e, and x = 1.6 was obtained for F-QSCE@30, which is lower than that of QSCE@30 (*x* = 1.9), indicating that the F segment hinders the coordination between TFSI^‒^ and Li^+^ driven by the polymer ester group [45]. Besides, a small value of *x* also implies low ion aggregates (Li^+^-TFSI^‒^-Li^+^) presented in the electrolyte, facilitating the Li^+^ transport [10] All these three factors demonstrate that introducing the F segments (F-QSCE@30) promotes the dissociation of lithium salt and reduces the formation of multi-ion aggregates (multiple Li^+^-TFSI^‒^), thereby enabling faster lithium-ion transport.

The local chemical environment of Li^+^ in F-QSCE@30 and QSCE@30 was also evaluated using the ^7^Li solid-state nuclear magnetic resonance (SNMR) spectroscopy. As illustrated in Fig. 1f, in the LiTFSI spectrum, a single prominent peak is detected at − 6.65 ppm, associated with the undissociated LiTFSI. In contrast, multiple peaks are displayed in the spectra of F-QSCE@30 and QSCE@30, indicating a more complex chemical environment for Li^+^. For QSCE@30, the ^7^Li SNMR spectrum was fitted with four peaks, representing different Li^+^ environments. Among these peaks, the light green peak located at − 12.9 ppm is the most dominant one, accounting for approximately 93.0% of the total signal. The spectrum of F-QSCE@30 was fitted with three peaks, and the purple one at − 13.2 ppm is the most significant, comprising about 87.4% of the total signal. The greater the main peak area, the more the Li^+^ is in a strongly bound state, and thus, introducing the fluorinated segments enhances the interaction between the fluorinated chains and Li^+^. In summary, both Ramana and ^7^Li SNMR demonstrated that the coordination environment of Li^+^ with TFSI^‒^ in F-QSCE@30 is different from that in QSCE@30. The inductive effect of F makes the electron cloud biased toward F, and the F surrounded by the electron cloud weakens the interaction between Li^+^ and TFSI^‒^, thereby reducing the amount of TFSI^‒^ around Li^+^ and increasing the dissociation degree of LiTFSI. Figure 1g illustrates the interaction between Li^+^ and ‒C = O‒O‒CF_2_‒ in the polymer. Due to the inductive effect, the electron cloud is biased toward the F atoms. The F atoms acquire a partial negative charge, while the carbon atoms assume a partial positive charge. Therefore, the F segment has a certain influence on the chemical environment and coordination of Li^+^.

### Electrochemical Properties and Performance

**Fig. 2:**
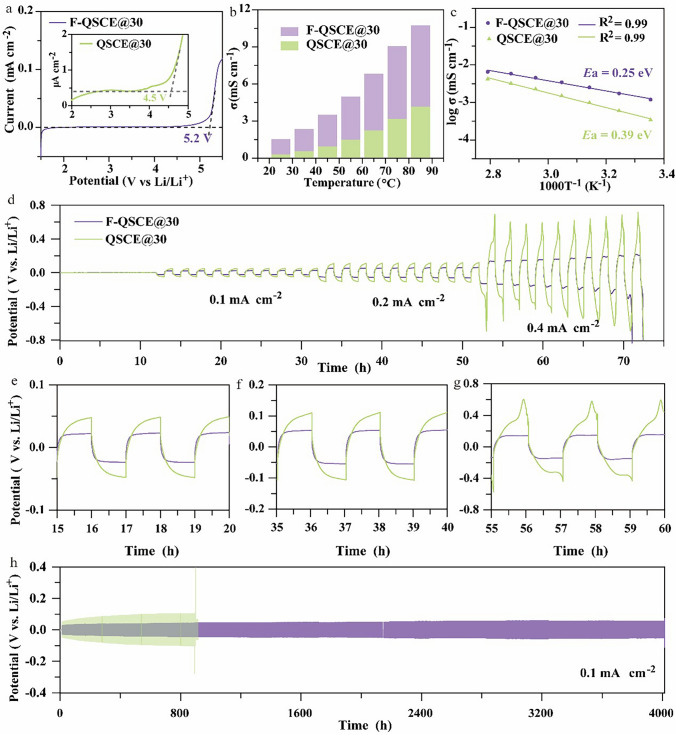
Electrochemical performances. **a** LSV at 25 °C. **b** Ionic conductivity at various temperatures. **c** Arrhenius plots. **d** Li plating/stripping performance of F-QSCE@30 and QSCE@30 at 0.1, 0.2, and 0.4 mA cm^−2^. Partially enlarged details of Li plating/stripping profiles, **e** 15–20 h at 0.1 mA cm^−2^, **f** 35–40 h at 0.2 mA cm^−2^, **g** 55–60 h at 0.4 mA cm^−2^. **h** Li plating/stripping performance of F-QSCE@30 and QSCE@30 at 0.1 mA cm^−2^

The LSV profiles were determined experimentally to assess the electrochemical stability window of the developed QSCEs. As depicted in Fig. 2a, F-QSCE@30 exhibits a high oxidation potential of approximately 5.20 V, while QSCE@30 shows an oxidation potential occurring at 4.50 V. Therefore, F-QSCE@30 demonstrates a broader electrochemical stability window compared to QSCE@30. The enhanced electrochemical stability of F-QSCE@30 is ascribed to the incorporation of the F segments, which effectively lowers the electron density around the oxygen atoms in the ester groups, making the oxygen atoms less susceptible to oxidation [43].

The ionic conductivities of F-QSCE@30 and QSCE@30 were measured from 25 to 85 °C. The results are illustrated in Fig. 2b. It demonstrates that both F-QSCE@30 and QSCE@30 display enhanced ionic conductivity as the temperature increases, while F-QSCE@30 consistently shows higher conductivity than QSCE@30. For example, F-QSCE@30 has an ionic conductivity of 1.21 mS cm^–1^ at 25 °C, whereas QSCE@30 demonstrates a lower ionic conductivity of 0.34 mS cm^–1^ at 25 °C. Subsequently, the activation energies (*E*a) of F-QSCE@30 and QSCE@30 were extracted from the temperature-dependent ionic conductivity, and they were found to be 0.25 and 0.39 eV (Fig. 2c), respectively, i.e., a lower Li^+^ transport barrier for F-QSCE@30 compared to QSCE@30. Therefore, the improved ionic conductivity of F-QSCE@30 is ascribed to the introduction of highly electronegative and polar fluorine atoms, which weakens the interaction between Li^+^ and TFSI^–^ by attracting and redistributing the electron clouds, thereby facilitating ion transport [43]. Meanwhile, the exceptionally high ionic conductivity of the F-QSCE is also due to its low Li^+^ transport barrier.

To further investigate the ion transport kinetics at the Li metal interface, the symmetric cells of Li/F-QSCE@30/Li and Li/QSCE@30/Li were assembled for evaluation, and the interfacial kinetics were analyzed using the Tafel plots. By fitting the Tafel curves, the exchange current density (*I*_0_) was determined, enabling an exploration of the Li^+^ transport dynamics at the lithium anode. As illustrated in Fig. S4, the* I*_0_ value for F-QSCE@30 reaches 0.031 mA cm^−2^, nearly 2 times higher than that of QSCE@30 (0.016 mA cm^−2^). The substantial increase demonstrates the improved Li^+^ charge transfer kinetics at the lithium metal interface, facilitated by F-QSCE@30. Figure S5 presents the investigation of *t*_Li_^+^ in F-QSCE@30 and QSCE@30 at 60 °C to assess the efficiency of the Li^+^ transport. F-QSCE@30 demonstrates a higher *t*_Li_^+^ than QSCE@30, with values of 0.41 for F-QSCE@30 and 0.34 for QSCE@30. A higher *t*_Li_^+^ indicates a more effective reduction in both the concentration polarization and the suppression of Li dendrite growth, leading to enhanced Li metal anode performance. Consequently, F-QSCE@30 shows promise for enhancing cell performance.

To investigate the electrochemical compatibility of the designed QSCEs with the Li metal, the cycling performance of the symmetric cells (Li/F-QSCE@30/Li, Li/QSCE@30/Li) was tested under different currents (0.1, 0.2, and 0.4 mA cm^–2^). As depicted in Fig. 2d, the cell using F-QSCE@30 displayed lower overpotentials compared to the one using QSCE@30. According to the partially enlarged details of the Li plating/stripping profiles at different currents presented in Fig. 2e–g, the deposition/stripping curves of F-QSCE@30 are smoother than those of QSCE@30, and the cell with F-QSCE@30 shows significantly lower overpotentials (F-QSCE@30: 23.0 mV vs. QSCE@30: 47.9 mV at 0.1 mA cm^–2^; F-QSCE@30: 55.7 mV vs. QSCE@30: 106.1 mV at 0.2 mA cm^–2^; F-QSCE@30: 147.4 mV vs. QSCE@30: 571.9 mV at 0.4 mA cm^–2^). Based on the previous electrochemical performance results, F-QSCE@30 demonstrates increased ionic conductivity and *t*_Li_^+^. Such improved performance indicates enhanced ion mobility and Li^+^ transport within the electrolyte, leading to reduced polarization effects and lower overpotential in the Li//Li cell [46].

As presented in Fig. 2h, the Li/F-QSCE@30/Li symmetric cell demonstrates a low overpotential of 24.2 mV and remains stable cycling for over 4000 h. This exceptionally good performance is ascribed to the formation of a stable SEI in the Li/F-QSCE@30/Li symmetric cell, which was further analyzed in the section on interface stability. In contrast, the Li/QSCE@30/Li demonstrates a high initial overpotential of 42.5 mV due to poor compatibility with the Li anode. Additionally, the overpotential of the cell with the QSCE@30 gradually rises during cycling and then sharply increases after 897 h with a short circuit, indicating that the interface with the lithium metal is unstable over cycling [47]. Besides, the long cycling of the Li/F-QSCE@30/Li cells at a current density of 0.2 mAh cm^–2^ (Fig. S6) shows stable voltage profiles over 900 h, indicating desirable interface stability and good electrochemical stability of F-QSCE@30 with the Li metal [48]. Thus, F-QSCE@30 is a favorable electrolyte for the Li metal anodes.

**Fig. 3:**
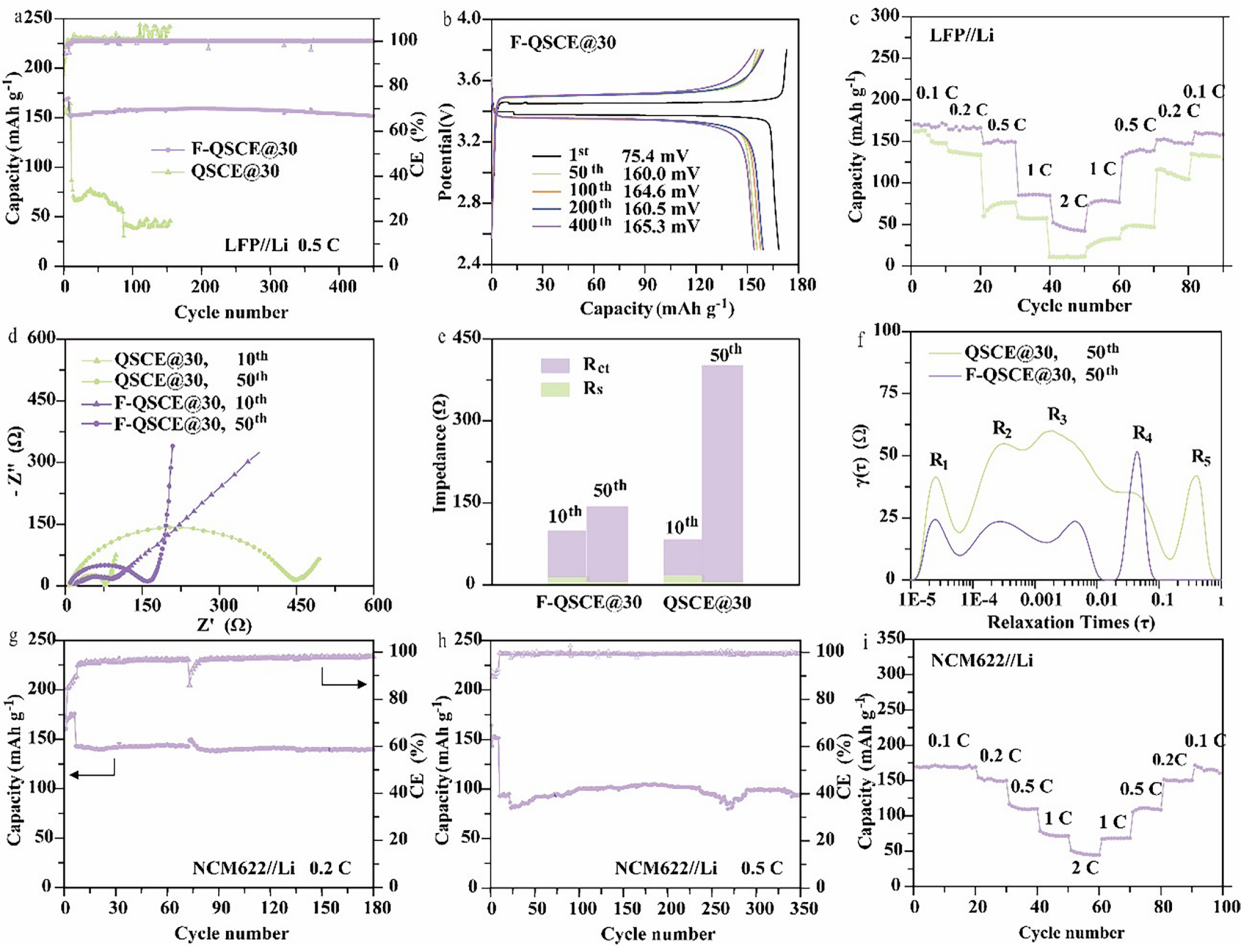
Electrochemical performance of the full cell in LMBs at 60 °C. **a** Cycle performance of LFP/F-QSCE@30/Li and LFP/QSCE@30/Li at 0.5C. **b** Charge and discharge curves of LFP/F-QSCE@30/Li cell at the 1st, 50th, 100th, 200th, and 400th cycles. **c** Rate performance of LFP/F-QSCE@30/Li and LFP/QSCE@30/Li. **d** Interface impedance of LFP/F-QSCE@30/Li and LFP/QSCE@30/Li after 10 and 50 cycles at 0.5C. **e** Corresponding fitting data of LFP/F-QSCE@30/Li and LFP/QSCE@30/Li after 10 and 50 cycles. **f** Distribution of relaxation time (DRT) of the Nyquist plots. Cycle performance of NCM622/F-QSCE@30/Li cell at **g** 0.2C and **h** 0.5C. **i** Rate performance of the NCM622/F-QSCE@30/Li cell (LiFePO_4_, 1C = 170 mAh g^−1^; NCM622, 1C = 170 mAh g^−1^)

F-QSCE@30 and QSCE@30 were further used in conjunction with both the LiFePO_4_ (LFP) and LiNi_0.6_Co_0.2_Mn_0.2_O_2_ (NCM622) in the electrochemical performance tests, and the cycling performance was also evaluated at 60 °C. As illustrated in Fig. 3a, the discharge capacity of the QSCE@30-based Li//LFP cell in the first cycle at 0.5C after activation is 86.2 mAh g^–1^, achieving a Coulombic efficiency of 98.7%. Due to the low ionic conductivity and potential interface issues of QSCE@30, a high discharge capacity cannot be performed. Furthermore, the cell exhibits a capacity retention rate of 53.2% after 150 cycles. In comparison, the LFP cell with F-QSCE@30 demonstrates a high initial discharge capacity and Coulombic efficiency (151.3 mAh g^–1^, 99.5%), along with an impressive capacity retention of 98.8% after 460 cycles. All these results indicate that F-QSCE@30 can maintain better cycling stability than QSCE@30.

The charge/discharge curves of the F-QSCE@30-based cell (Fig. 3b) indicate an initial polarization voltage of 75.4 mV at 0.1C, while the polarization voltages after 50, 100, 200, and 400 cycles at 0.5C are 160.0, 164.6, 160.5, and 165.3 mV, respectively, fluctuating within a small voltage range. For comparison, the cell based on QSCE@30 displays a higher polarization voltage of 77.4 mV at (1st cycle and 0.1C), 413.2 mV at (50th cycle and 0.5C), and 410.0 mV at (100th cycle and 0.5C) (Fig. S7). Therefore, F-QSCE@30 demonstrates a low polarization voltage, indicating fast Li^+^ transport in F-QSCE@30.

Rate performance is crucial in practical applications, and the charging time is associated with the transport efficiency of Li^+^ in the electrolyte [49]. Figure 3c highlights the rate performance of LFP/F-QSCE@30/Li and LFP/QSCE@30/Li cells across a range of 0.1C to 2C. The cell with F-QSCE@30 outperforms the one with QSCE@30, indicating more efficient Li^+^ transport within F-QSCE@30. The relatively poor rate performance of the full cells at 2C and 60 °C stems from interfacial instability, where the Li dendrite will form faster at high current densities (0.68 mA cm^−2^ at 2C, exceeding the 0.40 mA cm^−2^ in Fig. 2d). Furthermore, when the charge/discharge current decreases from 2 to 0.1C, the cell using F-QSCE@30 continues to demonstrate excellent reversible cycling, with a discharge capacity of 160.6 mAh g^–1^, which is much higher than 134.3 mAh g^–1^ for the cell using QSCE@30, being strongly linked to their ionic conductivity and *t*_Li_^+^.

The electrochemical impedance spectroscopy (EIS) was utilized to explore the behavior of the electrode/electrolyte interface in detail. As illustrated in Fig. 3d, the Nyquist plots of the various electrolytes show high-frequency semicircles and low-frequency linear regions, which correspond to the charge transfer resistance (*R*_ct_) and diffusion impedance (*R*_0_), respectively (Fig. S8), where the first intersection point at the *X*-axis (*Z*´) with semicircles corresponds to the intrinsic resistance (*R*_s_). The results of *R*_ct_ and *R*_s_ obtained from the EIS data (Fig. 3d) are shown in Fig. 3e. For the cells using F-QSCE@30 and QSCE@30, *R*_ct_ increases from the 10th to 50th cycle, attributed to the interface polarization and the development of an interfacial layer during cycling [50]. Further, after 10 cycles, their *R*_ct_ and *R*_s_ are comparable, while by the 50th cycle, the *R*_ct_ of the cell using F-QSCE@30 drops significantly compared to the one with QSCE@30 (F-QSCE@30, 136.5 Ω vs. QSCE@30, 412.9 Ω). The reduction in *R*_ct_ suggests differences in the electrode and electrolyte interface, which is reflected particularly by the morphology and composition [51]. The decreased *R*_ct_ in the F-QSCE@30-based cell highlights its superior interfacial stability [32].

The distribution of relaxation time (DRT) diagram is useful for disentangling the complex electrochemical processes that are interwoven in the Nyquist impedance diagram (Fig. 3d). As illustrated in Fig. 3f, in the LFP//Li cells, the overall polarization resistance is represented by three distinct peaks (*R*_1_, *R*_2_, and *R*_3_), while *R*_4_ and *R*_5_ correspond to the ion diffusion through the solid-state electrolyte. Each process is characterized by a time constant (*τ*), and the area beneath each peak reflects the polarization resistance of a specific electrochemical reaction, providing insight into the changes in both the property and extent of the electrode reactions [52]. Specifically, *R*_1_ corresponds to the polarization resistance at the electrode, primarily related to the Li^+^ transport across the interface layer, including cathode electrolyte interphase (CEI) and SEI, whereas *R*_2_ and *R*_3_ are linked to the charge transfer process [50]. The results reveal that the *γ*(*τ*) values of the *R*_1_, *R*_2_, and *R*_3_ peaks in the F-QSCE@30-based cell are smaller than those in the QSCE@30-based cell. A smaller peak area is generally indicative of faster interfacial processes, such as charge transfer or interfacial ion transport. Moreover, the ion diffusion peak in the F-QSCE@30-based cell manifests as a sharp spike, suggesting that the diffusion occurs within a narrow time frame, typically of a single rapid process. In contrast, the ion diffusion peaks (*R*_4_ and *R*_5_) in the QSCE@30-based cell appear as broader, dual peaks, indicating a longer timescale and suggesting a more complex diffusion process. These results demonstrate that F-QSCE@30 significantly enhances the interfacial dynamics and offers more efficient pathways for the Li^+^ transport.

The cycling performance of NCM622/F-QSCE@30/Li was further evaluated (Fig. 3g). The cell achieves a discharge capacity of 139.6 mAh g^–1^ after 180 cycles within a voltage from 3 to 4.3 V at 0.2C, achieving a high-capacity retention of 97.6%. Further analysis of the results shows that there is a simultaneous decline in the Coulombic efficiency and capacity surge at around the 70th cycle, which can be attributed to the formation and stabilization of the SEI layer. Around the 70th cycle, the SEI layer reaches a more stable state after undergoing initial formation and reorganization. During this process, the temporary decline in the Coulombic efficiency is caused by side reactions (e.g., electrolyte decomposition) and lithium loss associated with the SEI formation. Concurrently, the stabilization of the SEI layer improves the Li^+^ transport and enhances the electrode–electrolyte contact, leading to a transient capacity surge as more active material becomes accessible [32]. Moreover, when the current rate increases to 0.5C (Fig. 3h), the cell achieves a discharge capacity of 94.6 mAh g^–1^ after 350 cycles with a capacity retention close to 100%, with a capacity drop and raise in between. The observed capacity drops at the 230th cycle are likely due to the reduced electrolyte decomposition during long-term cycling at high voltage, while the capacity raises after the 260th cycle can be attributed to the activation of additional electrode material that is initially inaccessible [53].

The cell performance under high voltage was tested at different rates from 0.1 to 2C (Fig. 3i). It indicates that the cell exhibits discharge capacities of 169.6, 154.2, 116.4, 78.3, and 51.3 mAh g^–1^ at 0.1C, 0.2C, 0.5C, 1C, and 2C, respectively, delivering high discharge capacities under different currents. Notably, when the charge/discharge current is reduced from 2 to 0.1C, the discharge capacity of the cell recovers to 168.2 mAh g^–1^, very close to its initial capacity at 0.1C. The recovery to the near-initial capacity underscores the outstanding reversible cycling performance of the F-QSCE@30 electrolyte, demonstrating its potential for high-voltage applications.

### Exploring the Impact of F in F-QSCE@30

F-QSCE@30 was identified as a promising electrolyte, and the F segment was believed to be the contributor. To reveal the underlying mechanism, further studies were conducted in this part from the aspects of the chemical environment and coordination environment of Li^+^ and the role of F on Li^+^ and the F-linked carbon on TFSI^‒^. To compare and quantify these effects, the F in F-QSCE@30 was replaced by hydrogen (H) in the simulation software, denoted as H-QSCE@30; DFT was employed to evaluate the binding energy of Li^+^ in different sites with ‒C = O‒O‒ in both F-QSCE@30 and H-QSCE@30, as well as the distance of Li^+^ with the O in ‒C = O‒. The interaction and coordination number of Li^+^ with H-QSCEs@30 (‒C = O‒O‒, ‒C‒O‒C‒, and ‒CH_2_‒CH_2_‒CH_3_) and F-QSCEs@30 (‒C = O‒O‒, ‒C‒O‒C‒, and ‒CF_2_‒CHF‒CF_3_) were investigated by the MD simulations.

**Fig. 4:**
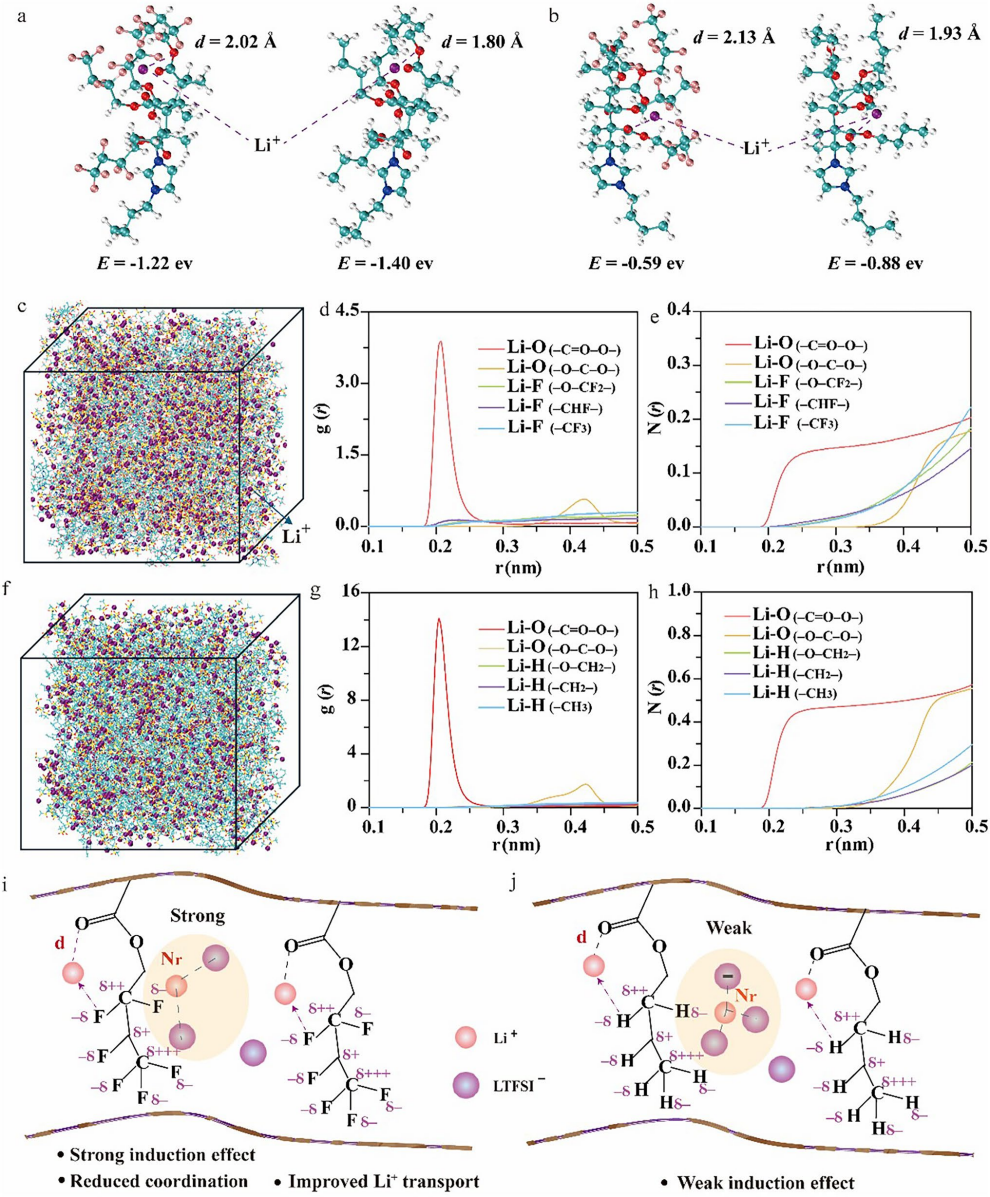
**a**–**b** Binding energy and distance of Li^+^ and ‒C = O‒O‒ in F-QSCE@30 and H-QSCE@30. **c** Three-dimensional snapshot of F-QSCE@30 system. **d** Radial distribution functions of Li^+^-O and Li^+^-F in F-QSCE@30. **e** Coordination number of Li^+^-O and Li^+^-F in F-QSCE@30. **f** Three-dimensional snapshot of QSCE@30 system. **g** Radial distribution functions of Li^+^-O and Li^+^-F in QSCE@30. **h** Coordination number of Li^+^-O and Li^+^-F in QSCE@30. Impact of **i** F‒C induction in F-QSCE@30 and **j** H‒C induction in QSCE@30

The binding energy of Li^+^ with the ‒C = O‒O‒ in F-QSCE@30 and H-QSCE@30 as well as the distance between Li^+^ and the O atom in ‒C = O‒ is shown in Fig. 4a, b. When Li^+^ is positioned as illustrated in Fig. 4a, the absolute value of the binding energy between Li^+^ and ‒C = O‒O‒ is lower in F-QSCE@30 (‒1.22 eV) than that of H-QSCE@30 (‒1.40 eV) [54]. The partial negative charge on the F atoms also interacts with Li^+^, thereby weakening the interaction between Li^+^ and ‒C = O‒O‒.

The weakening interaction promotes the Li^+^ transport and the dissociation of LiTFSI in F-QSCE@30. Additionally, the distance between Li^+^ and the O atom in ‒C = O‒ is 2.02 Å in F-QSCE@30. Compared to 1.80 Å in H-QSCE@30, it further indicates a reduced interaction between Li^+^ and ‒C = O‒O‒ in F-QSCE@30. Similar results are observed when the Li^+^ position is altered (Fig. 4(b); F-QSCE@30, ‒0.59 eV vs. H-QSCE@30, ‒0.88 eV; F-QSCE@30, 2.13 Å vs. H-QSCE@30, 1.93 Å).

Three-dimensional snapshots of F-QSCE@30 (Fig. 4c) and H-QSCE@30 (Fig. 4f) reveal that the introduction of the fluorinated segments enhances the ion dissociation and contributes to a more homogeneous distribution of Li^+^ [55]. The incorporation of fluorine alters the polymer chain configuration, thereby influencing the Li^+^ transport. To investigate this, the radial distribution function (RDF) was used to reflect the movement of Li^+^ along the fluorinated side chains in F-QSCE@30 and non-fluorinated side chains in H-QSCE@30 (Fig. 4d, g). The results show that, in F-QSCE@30 and H-QSCE@30, the peaks of Li‒O for the O atoms in ‒C = O‒ and that for the O atoms in ‒O‒C‒O‒ appear at 0.208 and 0.421 nm, respectively. The RDF peak intensities (g(r)) differ notably: In F-QSCE@30, the intensity at 0.208 nm is 3.86, which is much lower compared to 14.06 in H-QSCE@30; at 0.421 nm, the intensities are 0.56 (F-QSCE@30) and 1.73 (H-QSCE@30), respectively. A lower g(r) value in F-QSCE@30 indicates a weaker interaction between Li^+^ and the O atoms in the ‒C = O‒ and ‒O‒C‒O‒, facilitating the rapid transport of Li^+^ within F-QSCE@30. The weakened interaction is associated with the stronger interaction between Li^+^ and the partially negatively charged F [55]. Moreover, it is noteworthy that the H atoms on the side chains of H-QSCE@30 do not exhibit significant peaks with Li^+^, and the g(r) intensity is negligible, indicating that H has a minimal effect on Li^+^. This is attributed to the weak inductive effect resulting from the small electronegativity difference between C and H in the side chains of H-QSCE@30. Although the g(r) intensity between Li^+^ and the F atoms in F-QSCE@30 is also not prominent, a peak appears at 0.223 nm between Li^+^ and the F in ‒CHF‒, indicating that F does affect Li^+^, attributed to the strong inductive effect between F and C [16].

Figure 4e, h illustrates the coordination numbers of O and F on the fluorinated side chains of F-QSCE@30 with Li^+^, as well as the coordination numbers of O and H on the non-fluorinated side chains of H-QSCE@30 with Li^+^. In H-QSCE@30, the coordination numbers of Li^+^ with ‒C = O‒ and ‒O‒C‒O‒ on the side chains are 0.57 and 0.54, respectively, while in F-QSCE@30, these values are significantly reduced to 0.20 and 0.18. The weakened coordination number is conducive to the Li^+^ transport in the IL on the surface of SiO_2_@IL or in the polymer chains. Although there is no definite coordination number between Li^+^ and F (Fig. 4e) or H (Fig. 4h) on the side chains, the coordination between Li and F in F-QSCE@30 is notably enhanced, demonstrating a strong interaction between F and Li^+^, consistent with the RDF results [31].

Based on DFT and MD analyses, as well as previous characterization results, the strong induction effect of the C–F bond in F-QSCE@30 leads to a negative charge accumulation around the fluorine atom. This results in an enhanced electrostatic interaction with Li^+^, which in turn weakens the interaction between Li^+^ and the O atoms in the polymer. Moreover, the positively charged region around the C atom facilitates the immobilization of TFSI^‒^, further promoting the dissociation of LiTFSI and improving Li^+^ transport. This also helps prevent the aggregation of ion pairs (Li^+^-TFSI^‒^-Li^+^) (Fig. 4i). In comparison, H-QSCE@30 exhibits a weaker induction effect from the H-F bond, resulting in a less significant influence on the electrolyte properties (Fig. 4j).

### Interfacial Stability with Li

To further investigate the interfacial stability of F-QSCE@30 and QSCE@30 with the lithium metal and reveal the mechanism of the SEI formation, the morphology of the cycled Li metal anodes was characterized using SEM, and the SEI composition and its sources were revealed through XPS and ToF–SIMS in this part.

**Fig. 5:**
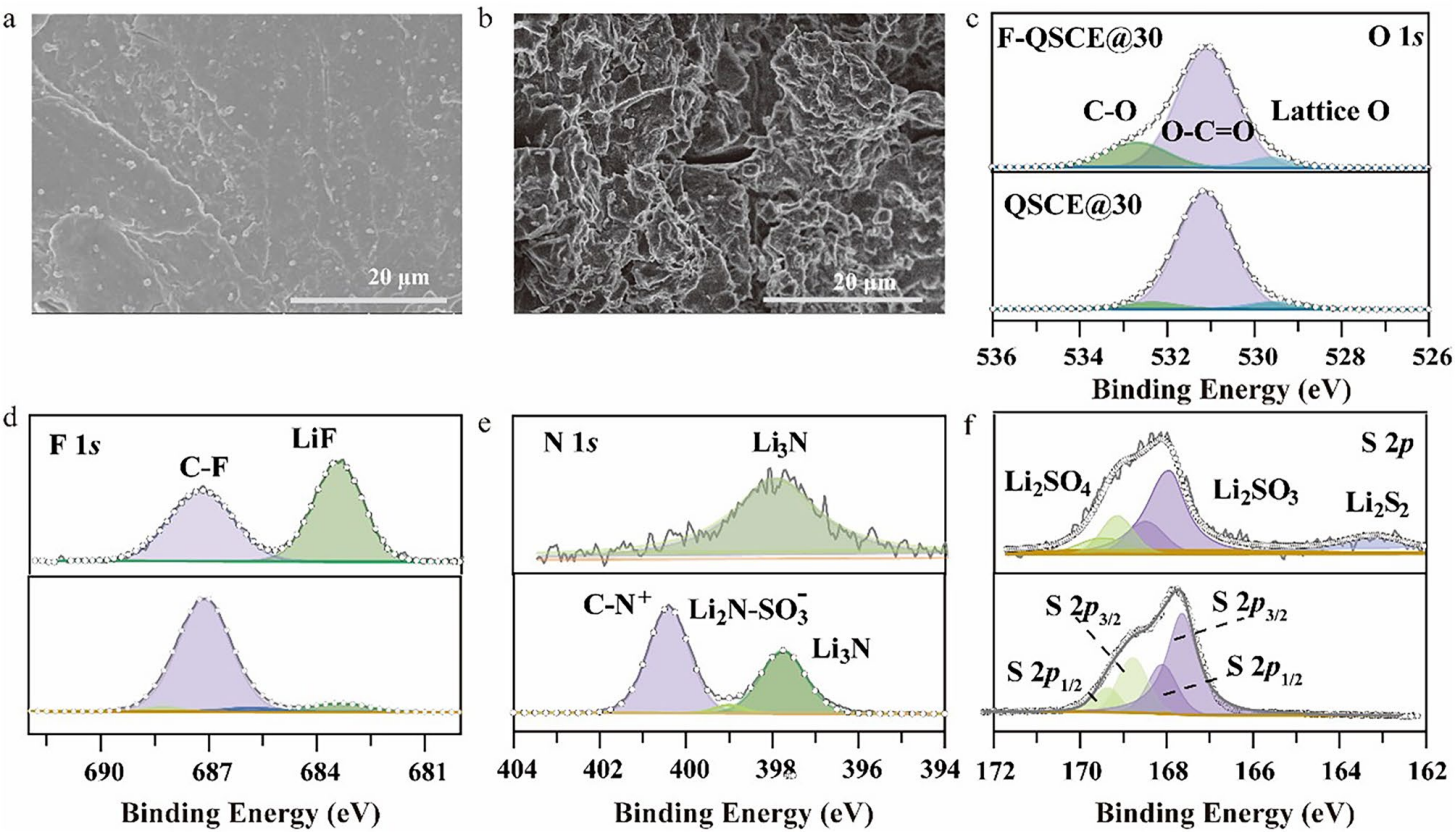
Investigation on the anode interface. The SEM images of cycled Li metal with **a** F-QSCE@30 and **b** QSCE@30 after 100 cycles. **c**–**f** XPS analysis of the Li metal surface after cycling, **c** O 1*s*, **d** F 1*s*, **e** N 1*s*, and **f** S 2*p*

The SEM images of the Li metal after 100 cycles, noted as the F-QSCE@30/Li and QSCE@30/Li interfaces, were obtained (Fig. 5a, b). As illustrated in Fig. 5a, at the F-QSCE@30/Li interface, the surface appears flat without dendrite growth, indicating uniform plating/stripping and confirming the improved Li^+^ transport capability of F-QSCE@30 [56]. However, the QSCE@30/Li interface shows noticeable cracks and a rough morphology (Fig. 5b), suggesting uneven Li^+^ transport and deposition [42]. The significant morphological differences between the two interfaces further highlight the importance and role of introducing the F segment.

To track the evolution of SEI, XPS was employed to study the composition of the Li metal anode surface after cycling. The O 1*s* spectra of F-QSCE@30/Li and QSCE@30/Li exhibit peaks corresponding to ‒O‒C = O (531.1 eV), C‒O (532.7 eV) [45], and lattice O (529.4 eV), suggesting the presence of organic oxides and Li_2_CO_3_ in the SEI of both samples (Fig. 5c) [48]. The F 1*s* spectrum of F-QSCE@30/Li displays a significantly high peak at 684.2 eV, corresponding to LiF (Fig. 5d) [57]; the intensity and area of the C-F peak on the Li metal surface are significantly weaker/lower than those of the LiF peak, indicating that the F-containing species in the SEI are predominantly inorganic LiF [28]. However, in QSCE@30/Li, the C-F peak intensity is notably stronger than that of LiF, implying that the fluorine-containing species in SEI are mainly organic. The N 1*s* spectrum peaks at 398.4 eV correspond to Li_3_N (Fig. 5e). The Li_3_N peak of QSCE@30/Li is notably weakened compared to that in F-QSCE@30/Li, and two new characteristic peaks appear, one is at 400.4 eV, linked to C-N^+^ from the pyrrolidine side chain of the copolymer or the cation of IL, and the other is at 399.1 eV, associated with Li_2_N-SO_3_^‒^ from the TFSI^‒^ decomposition [37]. The S 2*p* spectrum reveals the presence of Li_2_SO_4_, Li_2_SO_3_, and Li_2_S (163.2 eV) at the F-QSCE@30/Li interface (Fig. 5f), indicating a dominance of inorganic sulfides [58]. Conversely, in QSCE@30/Li, the peak associated with Li_2_SO_4_ and Li_2_SO_3_ is the most pronounced, while the intensities of Li_2_S (163.2 eV) are noticeably diminished [59], suggesting that the SEI also contains inorganic sulfides [37]. Based on the above analysis, it was found that the SEI in F-QSCE@30/Li is mainly composed of LiF, Li_3_N, and inorganic sulfides, whereas the SEI in QSCE@30/Li contains not only LiF, Li_3_N, and inorganic sulfides but also a significant amount of organic compounds.

Combining the results obtained in this work with the relevant literature, the formation mechanism of the SEI can be further explored (Fig. S9). When introducing the F segments (–CF_2_–CF–CF_3_), these fluorinated groups are preferentially reduced before TFSI⁻ due to their higher reactivity, which can generate small molecular fragments (e.g., –CF_2_, –CF–CF_3_) and ultimately form a LiF-rich interphase on the Li surface. The formation of this LiF-rich interphase has two key advantages: to suppress the TFSI⁻ reduction and improve interfacial stability. For the suppression of the TFSI⁻ reduction, the LiF-rich layer acts as a protective barrier, limiting the further reduction of TFSI⁻ at the Li interface. This suppression is beneficial because it reduces the decomposition of TFSI⁻ and minimizes the formation of undesirable by-products. Concerning the improvement of interfacial stability, the LiF-rich interphase enhances the mechanical and electrochemical stability of the anode interface, leading to better cycling performance and reducing the formation of lithium dendrite.

**Fig. 6:**
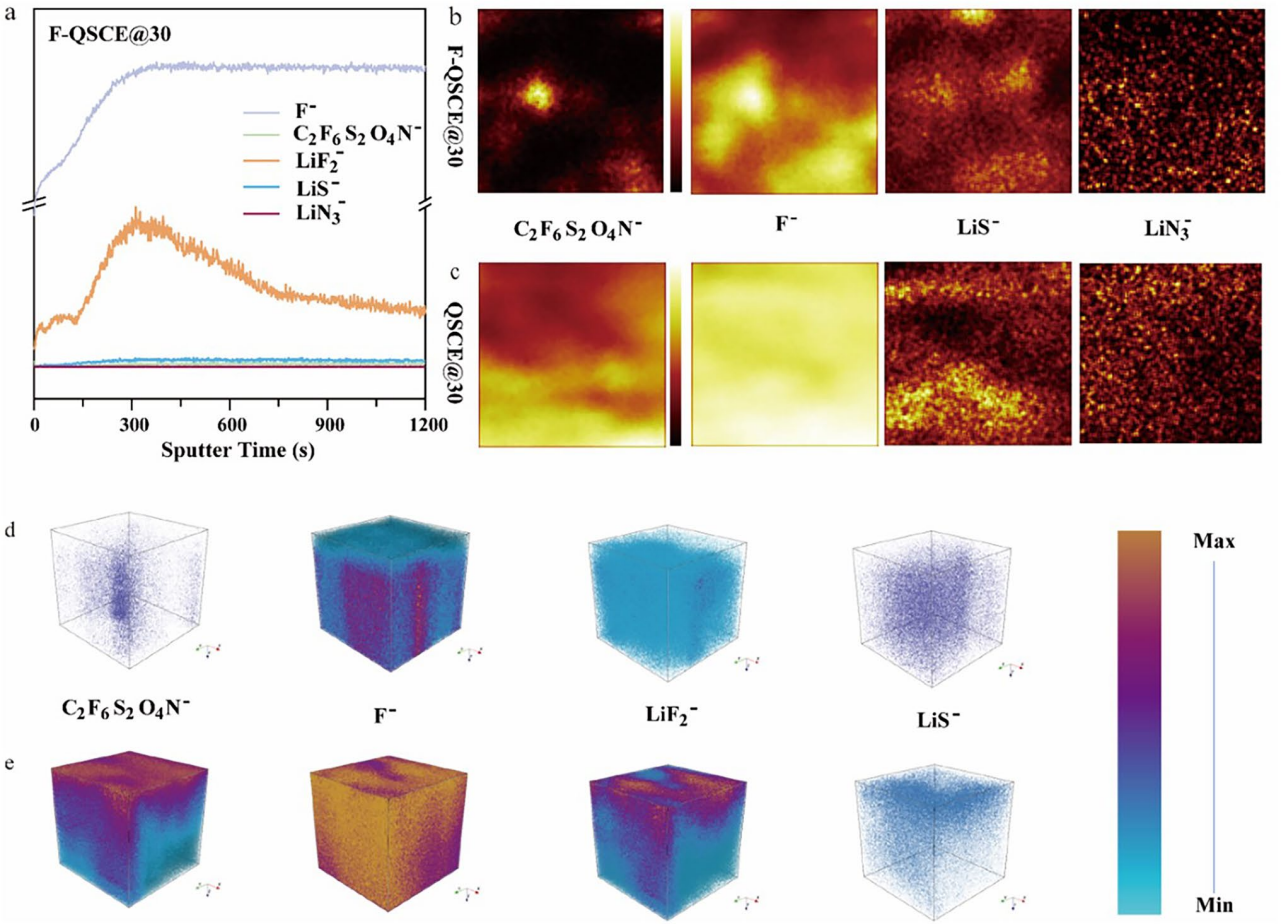
**a** Depth profiles of the secondary ions F^‒^, C_2_F_6_S_2_O_4_N^‒^, LiF_2_^‒^, LiS^‒^, and LiN_3_^‒^ at the interface between the Li metal and F-QSCE@30 in the LiFePO_4_//Li cell after 100 cycles. These profiles are normalized according to the peak intensities of each ion. The 2D mapping distribution of corresponding charged fragments in negative mode: **b** F-QSCE@30/Li and **c** QSCE@30/Li, analyzed from the lithium anode surface. Comparable 3D reconstructions of ToF–SIMS signals from the electrolyte decomposition products, including F^‒^, C_2_F_6_S_2_O_4_N^‒^, LiF_2_^‒^, and LiS^‒^ secondary ions: **d** F-QSCE@30/Li and **e** QSCE@30/Li. These 3D reconstructions visually depict the composition of the lithium interphase and their spatial distribution

To gain a more thorough understanding of the SEI composition and its distribution, ToF–SIMS was employed to characterize the distribution of ionic fragments on the lithium metal surface (Fig. 6a–e). In the LFP/F-QSCE@30/Li cell (Fig. 6a), the depth profiles obtained from ToF–SIMS reveal that the intensities of LiF_2_^‒^ and F^‒^ fragments are significantly higher than those of other fragments, while the intensities of C_2_F_6_S_2_O_4_N^‒^ and Li_3_N^‒^ fragments are much weaker, indicating that LiF in SEI mainly comes from the reduction of F segments [8]. In contrast, in the LFP/QSCE@30/Li cell (Fig. S10), LiF_2_^‒^ and C_2_F_6_S_2_O_4_N^‒^ fragments also exhibit much stronger intensities compared to the other fragments, with LiS^‒^ and Li_3_N^‒^ fragments being notably weaker. Since QSCE@30 lacks the dedicated F segments, the LiF presented in its SEI primarily originates from the reduction of TFSI^‒^ within the system. The high intensity of LiF_2_^‒^ highlights a relatively high proportion of LiF within the SEI in both cells. Furthermore, the SEI in the QSCE@30 cell contained a high proportion of organic compounds, consistent with the results from the XPS analysis.

Figure 6b, c shows the 2D surface mappings of the Li metal anode obtained in negative mode, demonstrating the uniform distribution of both organic fragments within the SEI of both cells. Notably, the lithium metal surfaces in both cells displayed a uniform and intense distribution of LiF, with the LFP/F-QSCE@30/Li cell also containing small amounts of LiC_2_F_6_S_2_O_4_N, Li_2_S, and Li_3_N [60]. By comparison, the LFP/QSCE@30/Li cell exhibited a higher intensity of LiC_2_F_6_S_2_O_4_N along with minor amounts of Li_2_S and Li_3_N.

The corresponding 3D mappings in Fig. 6d, e provide further insights, signifying that the signals of LiF_2_^‒^ and F^‒^ are strong, and there are few C_2_F_6_S_2_O_4_N^‒^ throughout the sputtering process on the Li surface of the LFP/F-QSCE@30/Li cell. Other uniformly distributed inorganic components, such as highly Li^+^-conductive LiS^‒^ and LiN_3_^‒^, contribute to the improved Li^+^ diffusion within the SEI. In the LFP/QSCE@30/Li cell, in addition to the strong signals of LiF_2_^‒^ and F^‒^, as the etching depth increases, there is also a stronger distribution of C_2_F_6_S_2_O_4_N^‒^, indicating the presence of more organic fluorinated components in the SEI. Meanwhile, it is worth noting that the 2D mapping (Fig. 6b, c) shows that the surface distribution of the F^‒^ species for the Li surface in QSCE@30/Li is more uniform than that of F-QSCE@30/Li, but the depth distribution of LiF_2_^‒^ and F^‒^ species is more consistent, as shown in the 3D reconstruction (Fig. 6d, e). This difference arises because, in the LFP/QSCE@30/Li cell, LiF mainly comes from the reduction of TFSI^‒^, resulting in a strong and uniform distribution of LiF on the Li surface. In contrast, in the LFP/F-QSCE@30/Li cell, LiF mainly forms through the reduction of -CF_2_-CF-CF_3_, resulting in poor surface uniformity, but promoting the formation of LiF throughout the SEI layer. Therefore, the LFP/QSCE@30/Li cell exhibits a more uniform surface distribution of LiF, while the LFP/F-QSCE@30/Li cell shows a better uniformity in the depth distribution of LiF species [61].

Combining the investigation on the Li^+^ chemical environment as well as the composition and distribution of the SEI, the mechanism of the inductive effect and the high interface stabilization of F-QSCE@30 in the NCM/Li cell was proposed. The strong electron-withdrawing ability of F in the –CF_2_–CF–CF_3_ group causes the electron cloud to shift toward F, which in turn strengthens the interaction between F and Li^+^. This shift also strengthens the interaction between the C atoms and TFSI^‒^, facilitating the dissociation of LiTFSI and reducing the coordination between Li^+^ and TFSI^‒^. Besides, in the presence of F segments (–CF_2_–CF–CF_3_), these fluorinated groups are preferentially reduced before TFSI⁻ due to their higher reactivity, generating small molecular fragments (e.g., –CF_2_, –CF_3_, –CF) and forming a LiF-rich interphase on the anode surface. This LiF-rich interphase suppresses the TFSI⁻ reduction by acting as a protective barrier, minimizing the electrolyte decomposition and the undesirable by-product formation. Thus, it enhances interfacial stability, improving overall cycling performance.

## Conclusions

In this work, a F-grafted QSCE (F-QSCE@30) was developed to improve the overall performance of the electrolyte, where the impact of the inductive effect of the F segments on the electrolyte performance as well as the influence of the F segments on the composition and formation of the SEI were investigated. For comparison, the fluorine-free electrolyte (F-QSCE@30) was prepared by replacing the fluorinated monomer with a fluorine-free monomer. The results demonstrate that F-QSCE@30 exhibits significantly improved performance of electrolyte and cell even compared with previously reported work (Table S2), including higher ionic conductivity of 1.21 mS cm^‒1^ at 25 °C, and more stable cycling for over 4000 h in the Li//Li symmetric cell, ascribed to the increased dissociation of LiTFSI, the weakening of the coordination between Li^+^ and TFSI^‒^, and the formation of a LiF-rich interface. Besides, the NCM622/F-QSCE@30/Li cell maintained nearly 100% capacity retention after 350 cycles at 0.5C. The DFT calculations and MD simulations highlighted that the inductive effect enhances the interaction between Li^+^ and F, as well as that between TFSI^‒^ and C, promoting the dissociation and uniform distribution of LiTFSI and weakening the coordination between Li^+^ and ‒C = O‒O‒. Furthermore, XPS and ToF–SIMS analyses confirmed that –CF_2_–CF–CF_3_ in the fluorinated polymer preferentially decomposes to form LiF over TFSI^‒^, contributing to the superior interfacial stability of F-QSCE. This work enhances the overall performance of QSCE while also offering valuable insights into the mechanisms driving these improvements, providing one more strategy for the development of high-performance QSCE.

## Supplementary Information

Below is the link to the electronic supplementary material.

Supplementary file1 (DOCX 808 KB)
