# Supplementary material for: Induction Effect of Fluorine-Grafted Polymer-Based Electrolytes for High-Performance Lithium Metal Batteries
Source: Nanomicro Lett. 2025 May 13;17:256. doi: 10.1007/s40820-025-01738-9 (PMC12075085; doi:10.1007/s40820-025-01738-9)
Supplement: Supplementary file 1 — Supplementary file1 (DOCX 808 KB) [file 40820_2025_1738_MOESM1_ESM.docx]

Supporting information for

**Induction Effect of Fluorine-Grafted Polymer-Based Electrolytes for High-Performance Lithium Metal Batteries**

Haiman Hu^1^, Jiajia Li^1^, Fei Lin^1^, Jiaqi Huang^1^, Huaiyang Zheng^1^, Haitao Zhang^2,^*, Xiaoyan Ji^1,^*

^1^ Energy Engineering, Division of Energy Science, Luleå University of Technology, Luleå 97187, Sweden

^2^ CAS Key Laboratory of Green Process and Engineering, Beijing Key Laboratory of Ionic Liquids Clean Process, Institute of Process Engineering, Chinese Academy of Sciences, Beijing 100190, P. R. China

*Corresponding authors. E-mail: [xiaoyan.ji@ltu.se](mailto:xiaoyan.ji@ltu.se) (Xiaoyan Ji); [htzhang@ipe.ac.cn](mailto:htzhang@ipe.ac.cn) (Haitao Zhang)

**S1 Experimental**

**S1.1 Characterization**

Fourier transformation infrared (FTIR) was obtained using a Thermo Nicolet 380 spectrometer, covering a range from 600 to 4000 cm^‒1^. The Raman spectra were measured with a HORIBA Scientific LabRAM HR Evolution, focusing on a wavenumber range of 700 to 900 cm^‒1^. The thermal stability was assessed through thermogravimetric analysis (TGA) with an STA7200RV instrument from Hitachi High-Tech. The differential scanning calorimetry (DSC) measurements were conducted using a Perkin Elmer Diamond apparatus, to determine the glass transition temperature (*T*_g_) across a temperature range of -90 to 25 °C, with a heating rate of 10 °C per minute. Additionally, ^7^Li NMR spectra were recorded on a Bruker AVANCE II 400 spectrometer, and X-ray photoelectron spectroscopy (XPS) was conducted with an ESCALAB 250Xi instrument. For the Time-of-flight secondary ion mass spectrometry (ToF-SIMS), the analysis was performed using a PHI nanoTOFII system, operated under vacuum conditions.

**S1.2 Electrochemical measurements**

The ionic conductivity was measured using symmetrical cells with stainless steel (SS) electrodes. The electrochemical impedance spectroscopy (EIS) was performed over a temperature range of 25 to 85 °C with a CHI660E electrochemical workstation, covering a frequency range from 0.01 to 1 000,000 Hz. The ionic conductivity (σ) was then calculated using the following equation:

$\sigma=\frac{L}{RS}$ (S1)

where *L* represents the thickness of the electrolyte, *R* is the resistance of the bulk electrolyte measured through EIS, and *S* denotes the area in contact with the 16 mm diameter stainless steel.

The Li^+^ transference number (*t*_Li_^+^) was determined through the potentiostatic polarization using a symmetric Li/QSCEs/Li cell at 60 °C. The EIS was performed over a frequency from 0.01 to 1,000,000 Hz with an amplitude of 20 mV. *t*_Li_^+^ was calculated by:

*t*_Li_^+^ = $\frac{I_{s}\left( \Delta V-I_{0}R_{0} \right)}{I_{0}\left( \Delta V-I_{s}R_{s} \right)}$ (S2)

where *I_0_* and *I*_S_ are the initial and steady-state currents, *R_0_* and *R*_S_ are the interface resistances of the cell before and after polarization, respectively, and Δ*V* represents the applied voltage.

The electrochemical stabilities of QSCEs were assessed through the linear sweep voltammetry (LSV). The asymmetric cell (SS/QSCEs/Li) underwent testing with a voltage sweep from 2.0 to 6.0 V at a scan rate of 0.1 mV·s^‒1^. The interfacial stability of the electrolytes to Li metal was evaluated using the symmetric Li//Li cells. The cycling performance for the LiFePO_4_/QSCE/Li and NCM622/QSCE/Li cells was tested using the LAND cell testing system (CT2001A LANHE). The cells were all exposed to a 60 °C oven for 12 h to enhance the interface fusion between the electrolyte and the cathode.

**S1.3 Computer simulations**

The **DFT calculations** were carried out by employing the Gaussian 16 software package. [S1] Geometry optimization and frequency analysis were executed utilizing the B3LYP/6-311G(d,p) level of theory, [S2, S3] with the DFT-D3BJ method by Grimme employed for the dispersion correction. [S4, S5] The single-point energy calculations were performed with M06-2X/def2tzvp level of theory. [S6, S7] A structural unit of F-QSCE and H-QSCE was considered to represent the polymer chain. The binding energy of Li (*E*_(binding)_) was calculated as Equation (S1) [S8]:

*E*_(binding)_ = *E*_(M+Li)_ ‒ *E*_(M)_ ‒ *E*_(Li)_ (S3)

Where *E*_(M+Li)_, *E*_(M)_, and *E*_(Li)_ denote the energies associated with F-QCSE-Li or H-QCSE-Li, F-QCSE or H-QCSE, and a single Li atom, respectively.

**Molecular dynamics** (MD) simulations were utilized to investigate the microstructure and interactions of the systems. The OPLS all-atom parameters [S9] (OPLS-AA) were used for F-QSCE, H-QSCE, and Li^+^, generated by web-based LigParGen [S10-S12]. The PYR_13_^+^ and TFSI^‒^ were modeled by the CL&P force field [S13, S14], which was compatible with OPLS-AA. The system consisted of 100 ILs, 1000 LiTFSI molecules, and 100 F-QSCE or H-QSCE molecules in each simulation.

The initial configurations for all the systems were prepared using the PACKMOL package. [S15] All the MD simulations were conducted in the NPT ensemble with the MD package GROMACS [S16] by the Parrinello-Rahman barostat [S17] at 1 bar with a coupling constant of 2 ps. The temperature was controlled through the V-rescale thermostat [S18] at 298 K with coupling constants of 200 fs. The equation of motion was solved with the leap-frog integration algorithm with a time step of 1 fs. The van der Waals term and real space electrostatic interactions were calculated via direct summation with a cutoff distance of 1.5 nm. The particle-mesh Ewald method [S19] was employed to handle long-range electrostatic interactions in reciprocal space, with an FFT grid spacing of 0.15 nm and an electrostatic energy tolerance of 10^-5^. Each simulation started with maintaining the system at 800 K for 5 ns, followed by annealing to 298 K over another 5 ns, and an additional 10 ns to reach equilibrium. A subsequent 10 ns production run was then conducted, with data collected at 1 ps intervals for analysis.

**Table S1** The ionic conductivity of electrolytes with different ratios of VBImTFSI/HFM (V/M) and content of LiTFSI

| Samples | Molar ratio  (V/M) | LiTFSI  (wt.% of V/M) | Photoinitiator  (wt.% of V/M) | σ (mS cm^‒1^) |
| --- | --- | --- | --- | --- |
| 1 | 1 | 20 | 2 | 0.013 |
| 2 | 2 | 20 | 2 | 0.004 |
| 3 | 3 | 20 | 2 | 0.012 |
| 4 | 4 | 20 | 2 | 0.028 |
| 5 | 5 | 20 | 2 | 0.023 |
| 6 | 4 | 20 | 2 | 0.110 |
| 7 | 4 | 30 | 2 | 0.596 |
| 8 | 4 | 40 | 2 | 0.231 |

**Table S2** Summary of the properties of different polymer-based electrolytes

| **Electrolyte** | **Tensile strength**  **(MPa)** | **σ**  **(S cm^−1^)** | **Li//Li**  **cycling** | **Initial specific capacity and Capacity retention** | **Refs.** |
| --- | --- | --- | --- | --- | --- |
| PTFEMA/PEO  /LiTFSI | 2.00 | 0.63 (80 °C) | 3800 h at  0.10 mA cm^−2^ and 70 °C | Around 100.0 mAh g^-1^ (LFP),  98% after 400 cycles at  70 mA g^-1^ and 70 °C | [S20] |
| P(IL-OFHDODA-VEC)/IL/LiTFSI | / | 1.37 (25 °C) | 2500 h at  0.10 mA cm^−2^ and 30 °C | 161.2 mAh g^−1^ (LFP),  87% after 600 cycles at 0.5 C and 30 °C | [S21] |
| TFE-alt-MEGVE)/LiTFSI | / | 0.27 (85 °C) | 2600 h at  0.05 mA cm^−2^ and 70 °C | Around 160.0 mAh g^−1^ (LFP),  100% after 50 cycles at 0.1 C and 85 °C | [S22] |
| C2-C9-3F  (6K)/LiTFSI | / | 0.02 (25 °C) | 1500 h at  0.20 mA cm^−2^ and 30 °C | / | [S23] |
| PEO-PVDF/LiTFSI-LLZTO/Li_2_ZrO_3_-CsPF_6_/2,4,6-TFA | / | 0.63 (25 °C) | 1100 h at  0.10 mA cm^−2^ and 30 °C | 149.7 mAh g^−1^ (LFP),  95% after 200 cycles at 0.5 C and 30 °C | [S24] |
| Co-polymer  /LiTFSI/LLZTO  /SN/FEC | / | 1.11 (25 °C) | 2000 h at  0.10 mA cm^−2^ and 30 °C | 172.4 mAh g^−1^ (NCM811),  61% after 400 cycles at 0.5 C and 30 °C | [S25] |
| LiSPNF membrane/  PEGDA:PETEA/  Liquid electrolyte | / | 1.36 (30 °C) | 200 h at  1.00 mA cm^−2^ and 30 °C | 143.2 mAh g^−1^ (LFP),  98% after 100 cycles at 1.0 C and 30 °C | [S26] |
| M-S-PEGDA/LiTFSI | 9.4 | 0.23 (25 °C) | 1600 h at  0.10 mA cm^−2^ and 40 °C | 143.7 mAh g^−1^ (LFP),  86% after 500 cycles at 0.5 C and 40 °C | [S27] |
| PNPU-PVDF-HFP/LiTFSI | 5.2 | 0.41 (30 °C) | 1000 h at  0.10 mA cm^−2^ and 30 °C | 150.0 mAh g^−1^ (LFP),  90% after 300 cycles at 0.2 C and 40 °C | [S28] |
| **F-QSCE** | **0.09** | **1.21 (25 °C)** | **4000 h at**  **0.1 mA cm^−2^ and 60 °C** | **151.8 mAh g^−1^ (LFP),**  **99% after 460 cycles at**  **0.5 C and 60 °C** | **This work** |

**Fig. S1** Ionic conductivity of polymer-based electrolytes with different molar ratios of IL/HFM

**Fig. S2** Ionic conductivity of polymer-based electrolyte with the optimized IL/HFM molar ratio (4:1) and different contents of Li salt


**Fig. S3** LSV of polymer-based electrolyte with the optimized IL/HFM molar ratio (4:1) and different weight contents of Li salt

**Fig. S4** Tafel plot for the Li/F-QSCE@30 and QSCE@30/Li symmetric cells at 60 °C

**Fig. S5** The chronoamperometry profiles and impedance spectra of F-QSCE@30 and QSCE@30 based on the Li//Li cells at 20 mV and 60 °C


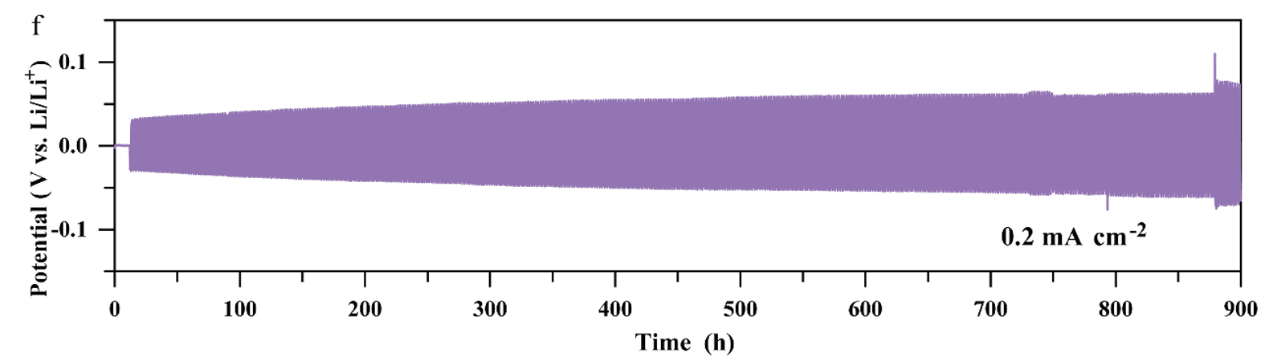


**Fig. S6** The Li plating/stripping profiles of F-QSCE@30 at 0.2 mA cm^−2^

**Fig. S7** Charge and discharge curves of LiFePO_4_/QSCE@30/Li cell at the first, 50^th^, and 100^th^ cycles


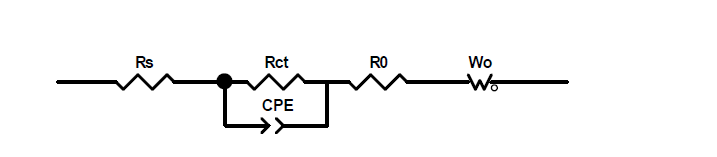


**Fig. S8** Equivalent circuit model of LiFePO_4_/F-QSCE@30 and QSCE@30/Li after 10 and 50 cycles at 0.5 C


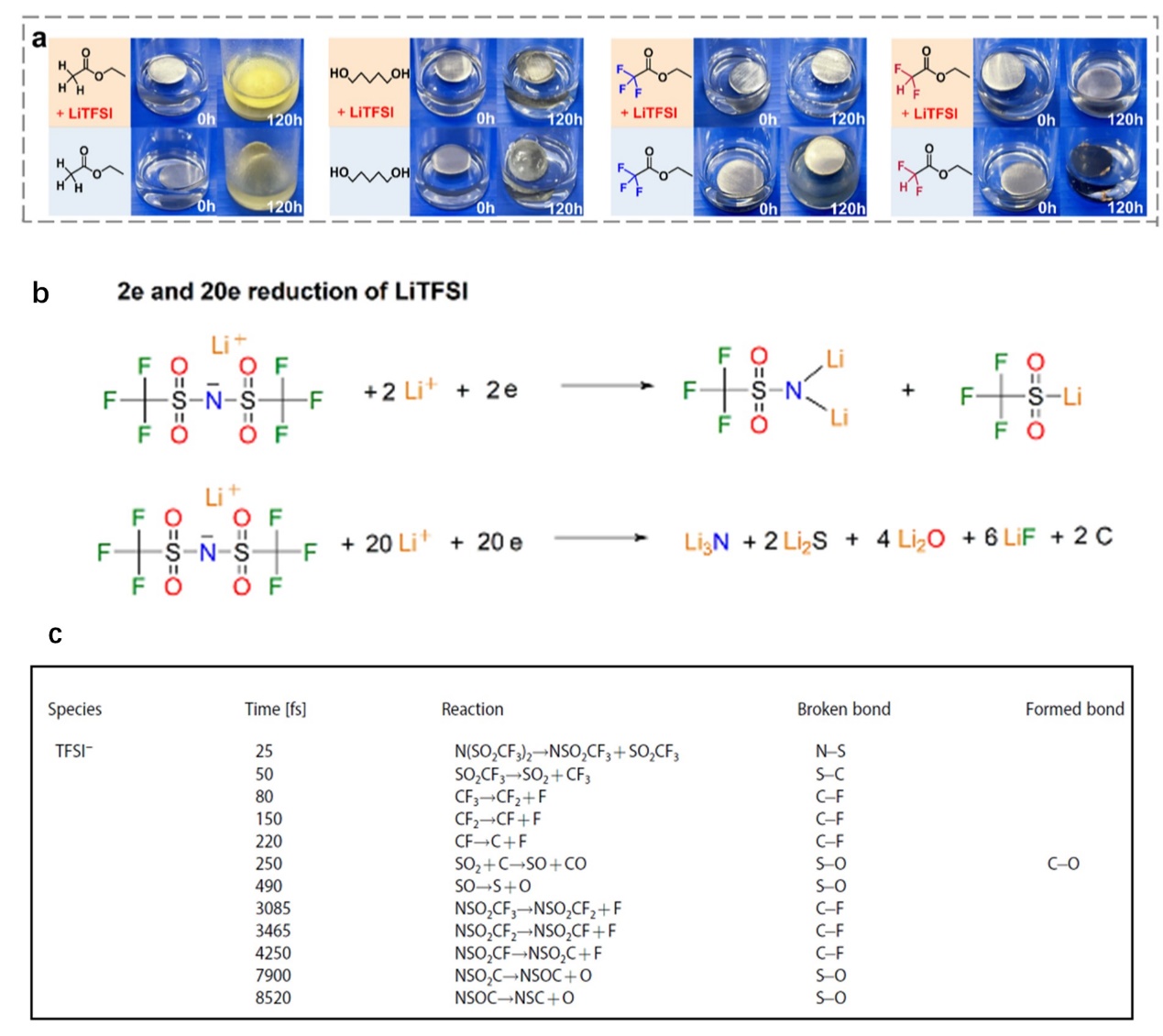
**Fig. S9** **a** Optical images of Li foil immersed in ethyl acetate, 1,5-pentanediol, ethyl trifluoroacetate, and ethyl difluoroacetate with and without LiTFSI for different times (0 and 120 h) at room temperature. (Copyright © 2024, American Chemical Society; J. Am. Chem. Soc. 2024, 146, 5940-5951) **b** Proposed mechanism for the reduction of LiTFSI. (Copyright © 2018, American Chemical Society; J. Am. Chem. Soc. 2018, 140, 9921−9933) **c** Catalog of electrolyte decomposition reactions and the approximate time they occurred during the AIMD simulations. (© 2022 Wiley‐VCH GmbH; Batteries & Supercaps 2022, 5, e202200088)

**Fig. S10** ToF-SIMS depth profiles with F^‒^, C_2_F_6_S_2_O_4_N^‒^, LiF_2_^‒^, LiS^‒^, and Li_3_N^‒^ after cycling in Li/QSCE@30/Li cell

**Supplementary References**

1. M.J. Frisch, G.W. Trucks, H.B. Schlegel, G.E. Scuseria, M.A. Robb et al., Gaussian 16 rev. C.01. (2016).
2. C. Lee, W. Yang, R.G. Parr, Development of the *Colle*-Salvetti correlation-energy formula into a functional of the electron density. Phys. Rev. B Condens. Matter **37**(2), 785–789 (1988). <https://doi.org/10.1103/physrevb.37.785>
3. R. Krishnan, J.S. Binkley, R. Seeger, J.A. Pople, Self‐consistent molecular orbital methods. XX. A basis set for correlated wave functions. J. Chem. Phys. **72**(1), 650–654 (1980). <https://doi.org/10.1063/1.438955>
4. S. Grimme, J. Antony, S. Ehrlich, H. Krieg, A consistent and accurate *ab initio* parametrization of density functional dispersion correction (DFT-D) for the 94 elements H-Pu. J. Chem. Phys. **132**(15), 154104 (2010). <https://doi.org/10.1063/1.3382344>
5. S. Grimme, S. Ehrlich, L. Goerigk, Effect of the damping function in dispersion corrected density functional theory. J. Comput. Chem. **32**(7), 1456–1465 (2011). <https://doi.org/10.1002/jcc.21759>
6. Y. Zhao, D.G. Truhlar, The M06 suite of density functionals for main group thermochemistry, thermochemical kinetics, noncovalent interactions, excited states, and transition elements: two new functionals and systematic testing of four M06-class functionals and 12 other functionals. Theor. Chem. Acc. **120**(1), 215–241 (2008). <https://doi.org/10.1007/s00214-007-0310-x>
7. F. Weigend, R. Ahlrichs, Balanced basis sets of split valence, triple *Zeta* valence and quadruple *Zeta* valence quality for H to Rn: Design and assessment of accuracy. Phys. Chem. Chem. Phys. **7**(18), 3297–3305 (2005). <https://doi.org/10.1039/b508541a>
8. Y.-F. Huang, T. Gu, G. Rui, P. Shi, W. Fu et al., A relaxor ferroelectric polymer with an ultrahigh dielectric constant largely promotes the dissociation of lithium salts to achieve high ionic conductivity. Energy Environ. Sci. **14**(11), 6021–6029 (2021). <https://doi.org/10.1039/D1EE02663A>
9. W.L. Jorgensen, D.S. Maxwell, J. Tirado-Rives, Development and testing of the OPLS all-atom force field on conformational energetics and properties of organic liquids. J. Am. Chem. Soc. **118**(45), 11225–11236 (1996). <https://doi.org/10.1021/ja9621760>
10. W. L. Jorgensen, J. Tirado-Rives. Potential energy functions for atomic-level simulations of water and organic and biomolecular systems. Proc. Natl. Acad. Sci. **102**(19), 6665-6670 (2005). <http://10.1073/pnas.0408037102>
11. L. S. Dodda, J. Z. Vilseck, J. Tirado-Rives, W. L. Jorgensen. 1.14* CM1A-LBCC: Localized bond-charge corrected CM1A charges for condensed-phase simulations. J. Phys. Chem. B **121**, 3864–3870 (2017). <https://doi.org/10.1021/acs.jpcb.7b00272>
12. L.S. Dodda, I. Cabeza de Vaca, J. Tirado-Rives, W. L. Jorgensen. LigParGen web server: An automatic OPLS-AA parameter generator for organic ligands. Nucleic Acids Res. **45**, 331-336 (2017). <http://doi/10.1093/nar/gkx312>
13. J.N. Canongia Lopes, J. Deschamps, A. A. Pádua. Modeling ionic liquids using a systematic all-atom force field. J. Phys. Chem. B. **108**, 2038-2047 (2004). <https://doi.org/10.1021/jp0362133>
14. J.N. Canongia Lopes, A.A. Padua, Molecular force field for ionic liquids III: imidazolium, pyridinium, and phosphonium cations; chloride, bromide, and dicyanamide anions. J. Phys. Chem. B **110**(39), 19586–19592 (2006). <https://doi.org/10.1021/jp063901o>
15. L. Martínez, R. Andrade, E.G. Birgin, J.M. Martínez, PACKMOL: a package for building initial configurations for molecular dynamics simulations. J. Comput. Chem. **30**(13), 2157–2164 (2009). <https://doi.org/10.1002/jcc.21224>
16. M.J. Abraham, T. Murtola, R. Schulz, S. Páll, J.C. Smith et al., GROMACS: High performance molecular simulations through multi-level parallelism from laptops to supercomputers. SoftwareX **1**, 19–25 (2015). <https://doi.org/10.1016/j.softx.2015.06.001>
17. M. Parrinello, A. Rahman, Polymorphic transitions in single crystals: a new molecular dynamics method. J. Appl. Phys. **52**(12), 7182–7190 (1981). <https://doi.org/10.1063/1.328693>
18. G. Bussi, D. Donadio, M. Parrinello, Canonical sampling through velocity rescaling. J. Chem. Phys. **126**(1), 014101 (2007). <https://doi.org/10.1063/1.2408420>
19. U. Essmann, L. Perera, M.L. Berkowitz, T. Darden, H. Lee et al., A smooth particle mesh Ewald method. J. Chem. Phys. **103**(19), 8577–8593 (1995). <https://doi.org/10.1063/1.470117>
20. Y. Su, X. Rong, A. Gao, Y. Liu, J. Li et al., Rational design of a topological polymeric solid electrolyte for high-performance all-solid-state alkali metal batteries. Nat. Commun. **13**(1), 4181 (2022). <https://doi.org/10.1038/s41467-022-31792-5>
21. L. Tang, B. Chen, Z. Zhang, C. Ma, J. Chen et al., Polyfluorinated crosslinker-based solid polymer electrolytes for long-cycling 4.5 V lithium metal batteries. Nat. Commun. **14**(1), 2301 (2023). <https://doi.org/10.1038/s41467-023-37997-6>
22. M. Ma, F. Shao, P. Wen, K. Chen, J. Li et al., Designing weakly solvating solid main-chain fluoropolymer electrolytes: synergistically enhancing stability toward Li anodes and high-voltage cathodes. ACS Energy Lett. **6**(12), 4255–4264 (2021). <https://doi.org/10.1021/acsenergylett.1c02036>
23. X. Xie, P. Zhang, X. Li, Z. Wang, X. Qin et al., Rational design of F-modified polyester electrolytes for sustainable all-solid-state lithium metal batteries. J. Am. Chem. Soc. **146**(9), 5940–5951 (2024). <https://doi.org/10.1021/jacs.3c12094>
24. T. Wang, B. Chen, C. Liu, T. Li, X. Liu, Build a high-performance all-solid-state lithium battery through introducing competitive coordination induction effect in polymer-based electrolyte. Angew. Chem. Int. Ed. **63**(16), e202400960 (2024). <https://doi.org/10.1002/anie.202400960>
25. A.-G. Nguyen, M.-H. Lee, J. Kim, C.-J. Park, Construction of a high-performance composite solid electrolyte through *in situ* polymerization within a self-supported porous garnet framework. Nano-Micro Lett. **16**(1), 83 (2024). <https://doi.org/10.1007/s40820-023-01294-0>
26. L. Wang, S. Xu, Z. Wang, E. Yang, W. Jiang et al., A nano fiber–gel composite electrolyte with high Li+ transference number for application in quasi-solid batteries. eScience **3**(2), 100090 (2023). <https://doi.org/10.1016/j.esci.2022.100090>
27. H. Wang, Q. Wang, X. Cao, Y. He, K. Wu et al., Thiol-branched solid polymer electrolyte featuring high strength, toughness, and lithium ionic conductivity for lithium-metal batteries. Adv. Mater. **32**(37), e2001259 (2020). <https://doi.org/10.1002/adma.202001259>
28. Y. Ye, X. Zhu, N. Meng, F. Lian, Largely promoted mechano-electrochemical coupling properties of solid polymer electrolytes by introducing hydrogen bonds-rich network. Adv. Funct. Mater. **33**(45), 2307045 (2023). <https://doi.org/10.1002/adfm.202307045>
